# Quality of life of the very old: Survey on quality of life and subjective well-being of the very old in North Rhine-Westphalia (NRW80+)

**DOI:** 10.1007/s00391-017-1217-3

**Published:** 2017-03-22

**Authors:** Michael Wagner, Christian Rietz, Roman Kaspar, Anna Janhsen, Luise Geithner, Michael Neise, Carolin Kinne-Wall, Christiane Woopen, Susanne Zank

**Affiliations:** 1 Faculty of Management, Economics and Social Sciences, Institute of Sociology and Social Psychology (ISS), University of Cologne, Cologne, Germany; 2 Faculty of Humanities, Work Area Research Methods, University of Cologne, Cologne, Germany; 3 Medical Faculty, Research Unit Ethics, University of Cologne, Cologne, Germany; 4 Faculty of Humanities, Rehabilitative Gerontology, University of Cologne, Cologne, Germany; 5 ceres – Cologne Center for Ethics, Rights, Economics, and Social Sciences of Health, 50931 Cologne, Germany

**Keywords:** Very old age, Quality of life, Welfare, Well-being, Population survey, Hochaltrigkeit, Lebensqualität, Lebensstandard, Wohlbefinden, Bevölkerungsumfrage

## Abstract

**Background:**

In Germany, the very old are the most rapidly growing proportion of the population. A comprehensive investigation of the conditions for a good quality of life in this group is relevant for both society and politics.

**Objective:**

The project “Quality of life and subjective well-being of the very old in North Rhine-Westphalia” (NRW80+) at the University of Cologne surveys quality of life of the very old. Taking into account many specific methodological and theoretical challenges, it aims at setting up a specific theoretical framework and methodological approach.

**Methods:**

Existing studies on quality of life in old age in Germany and abroad as well as models on quality of life are reviewed with respect to their relevance for the very old and their specific living conditions, needs and interests. A theoretical framework of quality of life in very old age is developed. The NRW80+ study combines three levels: the empirical level of description of life situations and conditions, the explanative level of evaluating models of quality of life and the normative level of societal and ethical standards and norms.

**Results:**

Considering results of recently conducted studies with the very old, an integrative conceptual model for studying quality of life of very old persons is introduced. In the model of challenges and potentials (CHAPO), environmental and individual factors as well as life chances and life results are thereby taken into consideration from a subjective as well as an objective point of view, supplemented by the concept of successful life conduct.

**Conclusion:**

Starting from the CHAPO model of quality of life, the representative study NRW80+ aims at challenging methodological standards for the inclusion of the very old in social research thus providing the basis for further research as well as for sustainable social politics especially for the very old.

## Zusammenfassung

**Hintergrund:**

Sehr alte Menschen stellen in Deutschland den am stärksten wachsenden Anteil an der Bevölkerung dar. Eine umfassende Untersuchung der Bedingungen für eine gute Lebensqualität dieser Bevölkerungsgruppe ist daher für Gesellschaft und Politik von besonderem Interesse.

**Ziel:**

Das an der Universität zu Köln angesiedelte Projekt „Lebensqualität und subjektives Wohlbefinden hochaltriger Menschen in NRW“ (NRW80+) untersucht die Lebensqualität der über 80-Jährigen in Nordrhein-Westfalen. Das spezifische theoretische und methodologische Design der Studie ergibt sich dabei aus der Berücksichtigung der zahlreichen methodologischen und theoretischen Besonderheiten, die eine Befragung dieser Bevölkerungsgruppe mit sich bringt.

**Methoden:**

Die bereits vorhandenen Studien zur Lebensqualität im Alter im deutschsprachigen Raum und auf internationaler Ebene sowie Modelle der Lebensqualität werden im Hinblick auf ihre Relevanz für Hochaltrige und deren spezifische Lebensbedingungen, Bedürfnisse und Interessen ausgewertet. Darauf aufbauend wird ein theoretisches Modell der Lebensqualität in der Hochaltrigkeit entwickelt. Die Studie NRW80+ berücksichtigt dabei 3 Reflexionsebenen: eine empirische Ebene der Deskription von Lebensvollzügen und -bedingungen, eine evaluative Ebene der Erklärung beobachtbarer Unterschiede in der Lebensqualität und eine normative Ebene der gesellschaftlichen und ethischen Standards und Normen.

**Ergebnisse:**

Aufbauend auf den Ergebnissen neuerer Studien über Hochaltrige wurde ein integratives Konzeptmodell zur Untersuchung der Lebensqualität hochaltriger Menschen vorgestellt. Das Modell der Challenges and Potentials (CHAPO) berücksichtigt Umwelt- und individuelle Faktoren sowie Lebensoptionen und -resultate sowohl aus subjektiver als auch objektiver Perspektive, ergänzt durch eine Dimension der gelungenen Lebensführung.

**Schlussfolgerung:**

Ausgehend vom vorgestellten CHAPO-Rahmenkonzept soll die NRW80+ Befragung bislang weitgehend ungenutzte Möglichkeiten für den Einschluss von Hochaltrigen in repräsentative Surveys aufzeigen und dadurch sowohl eine Grundlage für zukünftige Forschung als auch eine nachhaltige Sozialpolitik im Allgemeinen und Politik für ältere Menschen im Speziellen liefern.

## Background^1^

The substantial increase of population figures for the very old population requires among others the investigation of social, political, medical, nursing and ethical requirements of a good quality of life that enables subjective well-being [20, 22]. Quality of life is thus a broad concept that includes both objective and subjective indicators, ranging from the living conditions as an element of a persons’ environment to the disposable resources and evaluations of the personal life situation as a characteristic of individuals [35]. Furthermore, there is agreement that “quality of life” points to a relationship between an individual state as it is and a state as it should be [34]. In addition, both the individual’s evaluative stance and the external perspective of society that holds normative implications are to be considered [21]; however, a theoretical framework to investigate the quality of life of the very old that explicitly combines these descriptive, evaluative and normative perspectives has not yet been developed. Moreover, because of many serious methodological and practical problems in obtaining reliable and representative data, our knowledge about the life situation of the very old both in Germany and abroad is still limited; therefore, after briefly introducing the challenges and potentials (CHAPO) model of quality of life of the very old and giving a review of existing studies, this article outlines the theoretical and methodological design of the interdisciplinary project “Quality of life and subjective well-being of the very old in North Rhine-Westphalia” (NRW80+), which has recently been started at the University of Cologne. Thereby, it will also present some implications for scientific discourse and policy making.

## Quality of life of the very old

Approaches that integrate descriptive, evaluative, and normative perspectives in the discussion of quality of life in very old age are rare. Bringing together the three levels of empirical data, description of life situation and conditions, their subjective evaluation and their regulation by societal standards and norms, the NRW80+ study adopts a threefold perspective on quality of life in old age, which will be one of the unique features of this interdisciplinary study. In consequence, the definition of quality of life encompasses both objective and subjective approaches to quality of life and will contribute to a better understanding of the values and goals that guide older persons through late life. In addition, the results will be systematically reflected on the normative level [2, 33]. Based on a corpus of policy papers and qualitative interviews with stakeholders on topics of the prevailing societal discourse about the very old (e. g. costs in health care, economic and social potential as consumers and volunteers, impact of political effort on living conditions in the very heterogeneous group of older adults), we examine in which way the quality of life of very old people is influenced by normative stipulations or attitudes towards the very old population and motives underlying changes in age-critical infrastructure or political attention to aging issues. The perception of the very old regarding their recognition and position within society (e. g. feeling of anomaly) and social policies need to be taken into consideration as well.

To ascertain consistency of an interdisciplinary discussion of quality of life and to cover both life domains and processes of particular importance to subjective well-being and quality of life of the old and oldest old, the CHAPO conceptual framework is proposed. The model is based on Veenhoven’s [29] concept of “the four qualities of life” that distinguishes between “life chances” and “life results” on the one hand and “outer qualities” and “inner qualities” on the other hand. Following Veenhoven, although both are interrelated, “life chances” refer to opportunities, whereas “life results” describe outcomes, such as the life evaluation of the individual. “Outer qualities” refer to the environment of a person (e. g. infrastructure, social contacts) and “inner qualities” refer to the person’s characteristics (e. g. health, beliefs, competencies, education). Hence, this concept is a promising basis for the CHAPO framework because it distinguishes between various qualities and has already proved to be useful in interdisciplinary contexts [19, 29]; however, the CHAPO framework, differs from Veenhoven’s concept in a number of ways: firstly, it explicitly refers not only to environmental conditions in terms of opportunity structures but also to the system of goals and values both at the level of the person and the environment as a resource for or threat to quality of life. At the level of the environment, life chances include societal standards or values and the opportunities to realize individual goals and capacities. At the level of the individual person, life chances stand for individual standards, such as individual values or goals, as well as for actual conditions, such as disposable resources (e. g. financial, cultural, social), skills or competencies. In this way, the CHAPO framework supplements Veenhoven’s concept by delineating the patterns of environmental or individual values and resources.

Secondly, whereas person-environment (p-e) interactions are only indirectly mirrored by their satisfaction and well-being outcomes in previous approaches, the CHAPO framework explicitly seeks to delineate qualities of observed p‑e constellations. In doing so, the model offers a new flexibility to address quality of life outcomes beyond the hedonic realm. This includes functional [17] or eudemonic conceptions of quality of life [26] that are likely to be of particular relevance in very old age. The “Functional Quality of Life (fQOL)” model [17] considers the subjective evaluation of the individual’s resources to fulfil meaningful activities and goals. In our understanding, the conduct of activity may imply a grossly underestimated outcome quality, even if it does not (instantly) resolve in hedonic (e. g. wellbeing, happiness) quality of life outcomes.

**Fig. 1:**
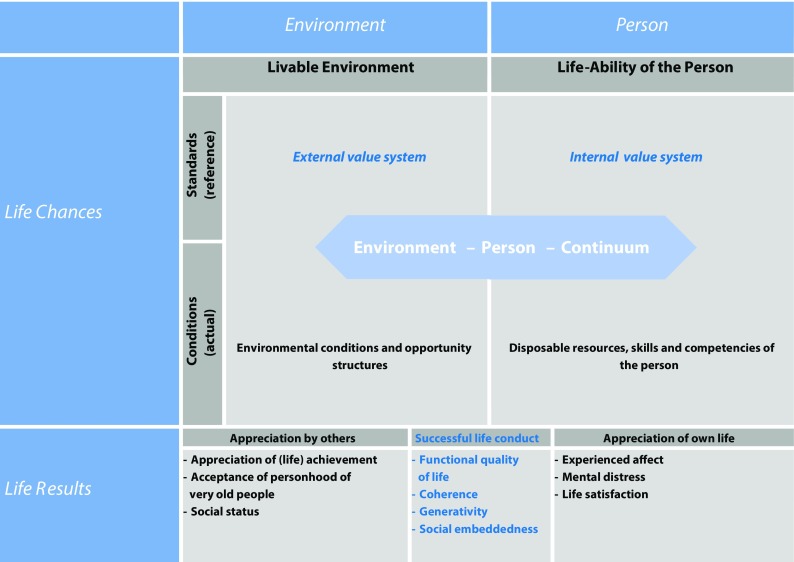
The challenges and potentials (CHAPO) model of quality of life in very old age. New conceptual elements are highlighted

Thirdly, again at the level of life results, the CHAPO framework not only distinguishes between appreciation by others and by the person. In addition, both perspectives are taken together and examined in terms of successful life conduct. From the perspective of society, e. g. a successful life conduct is a life that is useful for reaching collective goals or meeting societal standards. From the perspective of the person a successful life conduct results from personal development [26], life satisfaction or affective well-being. The fit of both perspectives makes up a successful life conduct in the sense of a functional environment-person continuum (Fig. 1).

## Studies of the very old in Germany and abroad

As a start, the databases PsycInfo, Psyndex, DIMDI, Cochrane, MEDPILOT have been searched by using the keywords very old age, quality of life and longitudinal study to identify existing international and national research projects including the population of the very old*. *In addition, we considered secondary internet-based literature and the Integrative Analysis of Longitudinal Studies on Aging Project (IALSA, http://www.ialsa.org/ [9.12.2015]) platforms. The results show that a number of studies in German language were designed to look specifically at old age (see Table 1). The Berlin Aging Study (BASE I) [15] investigated a locally representative sample from the general population at age 70+ years, with very old participants being overrepresented relative to their population counts [1]. The BASE I included a wide range of objective and subjective measures of the quality of life, covering social, financial, psychological, medical and clinical indicators; however, the regional focus of BASE as well as the still moderate number of very old participants constrain generalizations to the broader German population of very old persons. The subsequent BASE II study started a new panel in 2011 with 1600 participants aged between 60 and 80 years [6] and hence is not focusing on the very old. The *Generali Hochaltrigenstudie *[7] explores the perspective of 400 individuals aged 85 years and above using biographical interviews referring to life themes. Life themes correspond to the goals, orientations, needs and values of individuals. The respondents have been gathered through suggestions of welfare associations, churches, homes for the elderly, other institutions and recommendations, therefore the sample is highly selective. The two *Heidelberg Centenarian *studies [12, 25] have shown both the challenges and promises of population-based studies with the oldest-old. Naturally, the perspectives captured in these cross-sectional studies are those of late age survivors from a narrow range of respective birth cohorts, and as such may serve as a valuable reference for studying the quality of life in a broader segment in the old-old population. The *Austrian Interdisciplinary Study on the Oldest Old* (ÖIHS) has recently been piloted in the regions of Styria and Vienna. In the survey, a total of 410 residents aged between 80 and 85 years, living both in private and nursing homes have been considered [27]; therefore, it can serve as an example for the inclusion of very old people living in long-term care facilities even though the sample is relatively small and regionally focused. Also, very recently Conrad et al. [3, 4] conducted a population based study to standardize the WHOQOL-OLD instrument. As the emphasis is on validating an old age module to the existing WHO-QOL instrument its main focus is on subjective quality of life. The European project *Enabling Autonomy, Participation, and Well-Being in Old Age *(EnableAge) [9] contains a regionally focused population based German subsample of individuals living alone. The *Longitudinal Analysis of Subjective Well-being in Very Old Age* (LateLine) is a longitudinal follow-up of the German part of the EnableAge project. LateLine is focused on the influences of health constraints, the physical environment and anxiety concerning health and dying on subjective well-being and adaptation of the very old [31].

**Table 1:** German-speaking studies targeting the quality of life in the very old population

Study title	Age span (years)	Quality of life (selection of indicators)	Methodology	Reference
Berlin Aging Study (BASE I)	70+	Broad range of objective and subjective indicators	*N* = 516 (intensive protocol), random sample of West-Berlin residents, stratified by age and gender (1990/93)	[15]
Generali Hochaltrigenstudie (Interview study)	85–99	Themes of life (*Daseinsthemen*)	*N* = 400 In-depth biographical interviews, ad hoc sample (2013)	[7]
Heidelberg Centenarian Study I	100	Four aspects of quality of life: cognitive status, functional capacity, mental health, subjective well-being	*N* = 91, Local random sample (2000/01)	[25]
Heidelberg Centenarian Study II	100	Life satisfaction, meaning in life	*N* = 112, Local random sample (*n* = 95) (2011/13)	[12]
Austrian Interdisciplinary Study on the Oldest Old (ÖIHS)	80–85	Objective indicators: e. g. health, care, standard of living (quantitative study part); subjective indicators: e. g. life satisfaction, opinions (qualitative study part)	*N* = 410, Styria (150) and Vienna (260), local random sample; 40 qualitative interviews; (2013/14)	[27]
Quality of Life in the Elderly – Standardization of the WHOQOL-OLD	60+	Subjective quality of life (WHOQOL-BREF), six facets of the WHOQOL-OLD	*N* = 1133, German random sample, additional sample 80+ *(N =* 309) (2012)	[3, 4]
Enabling Autonomy, Participation, and Well-Being in Old Age: The Home Environment as a Determinant for Healthy Aging (EnableAge)	80–89	Home environment and housing, objective and subjective health, life satisfaction, affect	*N* = 450, German local random sub-sample (2002)	[9]
Longitudinal Analysis of Subjective Well-being in Very Old Age (LateLine)	87–97	Hedonic well-being (e. g. life satisfaction, affect), eudaemonic well-being (e. g. autonomy, purpose in life), mental distress	*N* = 124 (Baseline), German EnableAge follow up, seven measurement occasions from 2009/13	[31]

Most studies employ a broad understanding of quality of life spanning resources as well as outcomes, such as satisfaction with life. As a basis for our concept of quality of life in very old age, attention should be paid to the different methodological approaches and their implications for representativeness and selectivity as well as the comprehensiveness of the concept of quality of life. Whereas the former studies focused on the population of the very old, the *German Aging Survey* (DEAS) has been established as a national representative study in mid-life and old age. It is a valuable source for scientific investigation and policy making with respect to adults from age 40–85 years; however, since the DEAS is a population-based random sample, the absolute number of individuals aged 80 years and above in each of the 4 baseline samples to date (1996, 2002, 2008, 2014) do not enable a fine-grained analysis in this subpopulation [16].

On the international level as well, only a few population-based studies explicitly focus on the very old, including the EnableAge project conducted in Sweden, UK, Latvia, and Hungary [9], the *Georgia Centenarian *study [23], the *Fordham Centenarian* study [11], the *Swedish Panel Study of Living Conditions of the Oldest Old *(SWEOLD) [14], the *Newcastle 85+* study [5], and the *Swiss Interdisciplinary Longitudinal Study on the Oldest Old* (SWILSOO) [8]; however, the majority of these studies are not based on random samples from the general population. Moreover, the possibility to transfer policy implications that have been derived from these studies across countries, or even across states within countries are limited due to apparent differences in political administration, social security systems, infrastructure, and culture. Although a number of population-based studies include respondents aged 80 years and over, not all studies explicitly target quality of life in very old age [3]. Furthermore, a number of longitudinal studies have followed up their panel respondents up to their eighties and beyond. Among the 33 longitudinal studies on aging affiliated with the IALSA project, 14 studies did not originally include respondents aged 80 years and older at the first measurement occasion, and only a very limited number of studies exclusively targeted very old respondents from the beginning. Therefore, additional threats (e. g. panel mortality) to the generalizability of findings for these very old panel participants may need to be considered. Thus, all these different studies including the very old in Germany or abroad are either limited to certain regions, age segments or other types of selectivity, so that generalizable results for the very old in Germany cannot be derived; however, they all give important insights into different aspects of quality of life of the very old thus forming the knowledge base for the interdisciplinary project “Quality of life and subjective well-being of the very old in North Rhine-Westphalia” (NRW80+), which has recently started at the University of Cologne.

## Survey on quality of life of the very old: NRW80+

The overarching goal of the NRW80+ group is to improve the current state of research on the quality of life of very old people in Germany by taking additional efforts towards a representation of the very old in Germany’s most populated federal state, North Rhine-Westphalia (NRW). This includes substantial efforts to reduce selectivity in the sample. Furthermore, it strongly strives towards an interdisciplinary understanding of quality of life in old age. The survey will draw on objective social micro and macro conditions, subjective well-being, self-reported and tested health conditions and possibly objective biological markers as key instruments of the disciplines involved.

### Methodological complexity

As reported by the abovementioned studies, assessing subjective well-being and quality of life of the very old is a very demanding, time-consuming and costly effort, because it is vital to pay special attention to specific methodological challenges. Thus, a number of difficulties have been described regarding the inclusion of the very old in social sciences research and intervention studies [5, 13, 18].

First, some of the most vulnerable very old might not be reached under their registered address due to hospitalization, institutionalization or death. Even for older adults with less compromised health status, successful recruitment involves, among other things, additional time and effort [24]. In the cognitive impaired elderly, informed consent procedures often involve a legal representative to decide on study participation. For persons living in long-term care facilities, management and care staff have been described as additional gatekeepers [10]. Similar problems with regard to under-representation can be expected for individuals with a substantial need for care in home care settings, especially in constellations characterized by high caregiver burden. In consequence, the majority of large-scale survey studies are restricted in their possibilities to represent the more vulnerable, less accessible segments of the old and very old population.

Second, even after successful identification and inclusion of study participants, a number of threats to the validity of the information obtained have been described. Most importantly, the heterogeneity observed in the young-old population has not disappeared in very old interview partners, even if the physical and psychological vulnerability has increased substantially, and assessment procedures and coding schemes will struggle to address this diversity. In addition, loss of sensory function, such as hearing problems may be a major obstacle in the interview setting. In addition to the consequences of adverse physical conditions, explorations of quality of life of very old persons need to acknowledge the emotional vulnerability in this group. The high prevalence of depressive symptoms in very old age make interviews on life review and appraisal a very demanding endeavor [32].

### Study design

To overcome the aforementioned content-related and methodological problems, the NRW80+ builds on the results of a preceding feasibility study in defining the optimal sampling strategy, the approach of potential respondents and the assessment protocol. Using personalized advance letters and computer-administered personal interviews, a first branch addresses the conditions that allow for an inclusion of vulnerable and hard to reach older adults in a random sample of 1800 individuals drawn from registration offices of 6 communities in NRW. The study was approved by the ethics committee of the Medical Faculty of the University of Cologne. Informed consent was obtained from all participants. First results show unexpectedly high levels of reachability and survey participation. For example, based on the American Association for Public Opinion Research standard definitions [28], the cooperation rate was 40.5% and response rate was 26.5%. Multiple logistic regressions revealed that gender, age group and living in an old age home do not significantly affect the likelihood of nonresponse (OR = 0.98–1.32 n. s.). Nevertheless, adequate representation of the oldest old is only possible by including proxy informants.

A second branch uses a mixed-methods (e. g., cognitive interviews), multiformat (e. g., senior university classes) approach to pilot and revise all survey materials that will be implemented in the NRW80+ main study. The NRW80+ main study is designed with the aim of augmenting the DEAS in capturing the very old age segment of the population in NRW. To the extent that the different research foci allow, measures are harmonized to allow for an optimal cross-referencing of study results. The representative study will include up to 1800 realized interviews with persons aged 80 years and older, living in both private homes and institutional settings or proxy informants. Older persons (85+ years) and men will be overrepresented in the register sample to allow for detailed analyses in subpopulations of the very old. Regional disparities will be investigated based on a substantial sample of 94 communities with different historical and sociostructural characteristics.

## Conclusion

Establishing valid and reliable insights into the quality of life and subjective well-being in the subpopulation of the very old is a challenging endeavor, and the NRW80+ study aims to break new grounds in multiple domains. The general goals of the study are threefold: first, by introducing the CHAPO model of quality of life the study proposes a general model of quality of life sensitive to resources and aims in late phases of the life course. This involves a comprehensive description of the quality of life of the very old, an evaluation and explanation of the different qualities of life and a normative perspective that links societal standards and values as well as ethical norms and political measures with the quality of life of the very old.

Second, the establishment of methodological standards for the inclusion of the very old in social research requires minimizing sampling and non-response bias in order to get valid parameter estimates of the population of the very old. Apart from conducting the described feasibility study, survey instruments have to be developed that adapt to the health limitations of the very old living in private households and old age homes.

Third, by also taking up the question of self-determined living and the necessary social framing of old and oldest age, the NRW80+ contributes to the current senior citizen policy of NRW. This policy is committed to enabling old and very old citizens to live their lives in a self-determined manner, preferably in the middle of society. Not much, however, is known about the impact of social inequalities, such as social exclusion or even discrimination, on quality of life in this segment of the population [30]. The relevance of social inequalities on health and living conditions (e. g. level of accommodation and housing, activities and social support) need to be taken into account for future policy making.

Hence, in the study NRW80+, older people are, on the one hand given a voice in the fundamental discourse on aging and quality of life in old age. Their individual views on life, as well as their needs and wishes (e. g. regarding social policy) are the foundation of the study. On the other hand, senior citizen policy will be provided with promising and necessary measures to improve the quality of life especially in the later phases of the life course.

## Footnotes

1 The project NRW80+ belongs to the Key Research Area “Aging and Demographic Change” (Speaker: Susanne Zank) of the Cologne Center for Ethics, Rights, Economics, and Social Sciences of health (ceres) which is directed by Christiane Woopen at the University of Cologne. Members of the project board are Christian Rietz, Michael Wagner, Christiane Woopen and Susanne Zank. NRW80+ is funded by the Ministry of Innovation, Science and Research, North Rhine-Westphalia.
